# Production, characterization and determination of the real catalytic properties of the putative ‘succinate dehydrogenase’ from *Wolinella succinogenes*

**DOI:** 10.1111/j.1365-2958.2008.06581.x

**Published:** 2009-01-16

**Authors:** Hanno D Juhnke, Heiko Hiltscher, Hamid R Nasiri, Harald Schwalbe, C Roy D Lancaster

**Affiliations:** 1 Cluster of Excellence ‘Macromolecular Complexes’, Max Planck Institute of Biophysics, Department of Molecular Membrane BiologyMax-von-Laue-Str. 3, D-60438 Frankfurt am Main, Germany; 2 Cluster of Excellence ‘Macromolecular Complexes’, Institute of Organic Chemistry and Chemical Biology, Center for Biomolecular Magnetic Resonance, Johann Wolfgang Goethe UniversityMax-von-Laue-Str. 7, D-60438 Frankfurt am Main, Germany; 3 Department of Structural Biology, Faculty of Medicine, Saarland UniversityD-66421 Homburg (Saar), Germany

## Abstract

Both the genomes of the epsilonproteobacteria *Wolinella succinogenes* and *Campylobacter jejuni* contain operons (*sdhABE*) that encode for so far uncharacterized enzyme complexes annotated as ‘non-classical’ succinate:quinone reductases (SQRs). However, the role of such an enzyme ostensibly involved in aerobic respiration in an anaerobic organism such as *W. succinogenes* has hitherto been unknown. We have established the first genetic system for the manipulation and production of a member of the non-classical succinate:quinone oxidoreductase family. Biochemical characterization of the *W. succinogenes* enzyme reveals that the putative SQR is in fact a novel methylmenaquinol:fumarate reductase (MFR) with no detectable succinate oxidation activity, clearly indicative of its involvement in anaerobic metabolism. We demonstrate that the hydrophilic subunits of the MFR complex are, in contrast to all other previously characterized members of the superfamily, exported into the periplasm via the twin-arginine translocation (tat)-pathway. Furthermore we show that a single amino acid exchange (Ala86→His) in the flavoprotein of that enzyme complex is the only additional requirement for the covalent binding of the otherwise non-covalently bound FAD. Our results provide an explanation for the previously published puzzling observation that the *C. jejuni sdhABE* operon is upregulated in an oxygen-limited environment as compared with microaerophilic laboratory conditions.

## Introduction

A major obstacle to the exploitation of the large volume of genome sequence data is the functional characterization of the gene products (Wild and Saqi, 2004). Annotation is normally inherited from database matches to similar sequences for which the function is known and is hence prone to error propagation. Consequently, the number of functionally characterized gene products has to be increased, especially for those which are not easily accessible, as is the case for proteins that are produced in only small or non-detectable amounts under currently known laboratory growth conditions.

Previous determination of the genome sequence of the anaerobic epsilonproteobacterium *Wolinella succinogenes* surprisingly revealed that it encodes for protein complexes annotated to be part of an aerobic respiratory chain (Baar *et al*., 2003). Among these is a protein complex ostensibly involved in aerobic respiration and annotated to be a non-classical succinate:quinone reductase (SQR) which has so far not been detected under currently known growth conditions. A similar enzyme is encoded by the *Campylobacter jejuni* genome (Parkhill *et al*., 2000). SQRs are membrane protein complexes that couple the two-electron oxidation of succinate to fumarate to the reduction of quinone to quinol (Lancaster, 2004). Together with the quinol:fumarate reductases (QFRs) of anaerobic fumarate respiration, which catalyse the reaction in the reverse direction *in vivo*, the SQRs form the superfamily of succinate:quinone oxidoreductases (SQORs) (Hägerhäll, 1997; Lancaster, 2002). They generally contain three or four subunits: a flavoprotein (subunit A), an iron–sulphur protein (subunit B) and one large or two small membrane anchor subunits, referred to as subunit(s) C (and D). Subunit A contains the dicarboxylate oxidation/reduction site; the quinone/quinol binding site is located in the hydrophobic subunit. Among species, the hydrophilic subunits A and B have high sequence identity, while that for the membrane anchor is much lower. Based on their hydrophobic subunit and haem content SQORs can be classified in five types established as A, B, C, D or E in the literature (see Hederstedt, 1999; Lancaster, 2002 for reviews). Both type A and B enzymes contain two haem groups, the latter possesses one large hydrophobic subunit instead of two small ones as is the case for all the other types. Type C enzymes harbour a single haem, D-type and E-type enzymes contain no haem at all. Over the past nine years, three-dimensional structures of representatives of B-type (Lancaster *et al*., 1999; Madej *et al*., 2006a), C-type (Yankovskaya *et al*., 2003; Sun *et al*., 2005; Huang *et al*., 2006) and D-type (Iverson *et al*., 1999; Iverson *et al*., 2002) members of the superfamily have become available. The hydrophobic subunits of the E-type SQORs are very different from the other four types. They are more similar to those of heterodisulphide reductases from methanogenic archaea. Non-classical (‘E-type’) SQORs are not well characterized; the so far best-characterized enzyme is the SQR from the crenarcheon *Acidianus ambivalens* (Lemos *et al*., 2002; Schäfer *et al*., 2002). To our knowledge no genetic manipulation of an operon encoding for a ‘non-classical’ SQOR has been reported.

**Fig. 1:**
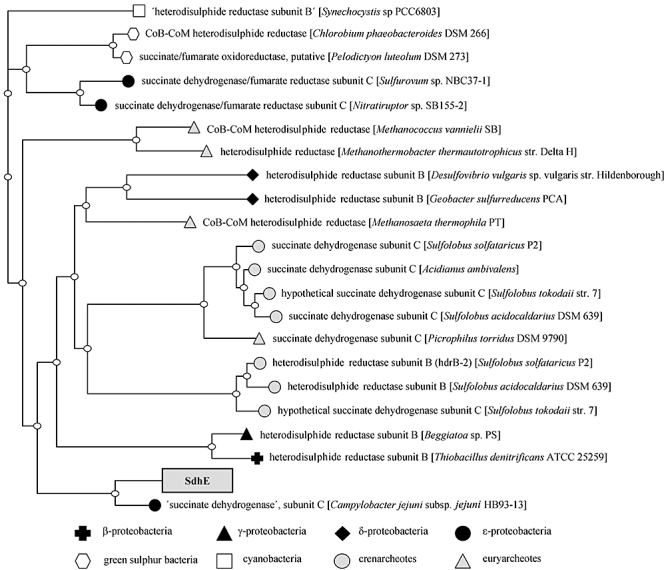
The phylogenetic relationship of the unusual membrane anchor subunit SdhE to selected organisms. A similar enzyme complex is encoded by the *Campylobacter jejuni* genome. The *W. succinogenes sdhE* gene (locus tag WS1022; see Fig. 2A) is annotated as *sdhC*; it was renamed to *sdhE* in analogy to the *Acidianus ambivalens* homologue (Lemos *et al*., 2002). The gene product was named accordingly SdhE.

The putative SQR from *W. succinogenes* consists of the two hydrophilic subunits SdhA, SdhB and a hydrophobic subunit SdhE which are encoded by the *sdhABE* operon (Fig. 1). The subunits SdhA, SdhB and SdhE show the highest sequence identity to the putative SQR from *C. jejuni*, of 63%, 71% and 69%, respectively, and of 38%, 27% and 32%, respectively, to the *A. ambivalens* enzyme. The similarity to the cyanobacterial enzyme subunits from *Synechocystis* is 37% for SdhA, 29% for SdhB1, 25% for SdhB2 and 40% for the putative ‘heterodisulphide reductase subunit b’ which could possibly be the membrane subunit for an SdhABE complex. The SQR from *A. ambivalens* contains a second hydrophobic subunit SdhF but no similar protein is encoded by the *W. succinogenes* and *C. jejuni* genomes.

**Fig. 2:**
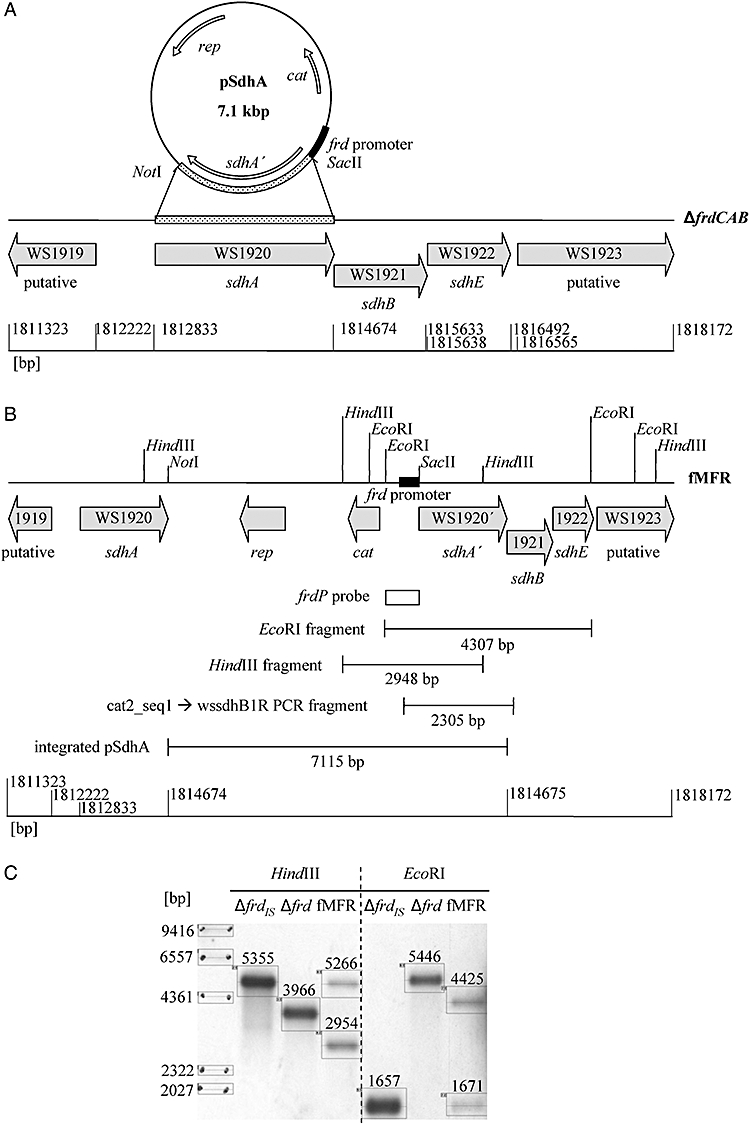
Genetic system for the production and manipulation of the SdhABE complex. A. In the vector pSdhA the structural gene *sdhA* is cloned next to the strong *frd* promoter (black box). The vector integrates via the homologous *sdhA* gene (dotted boxes) at the native locus in the genome of the QFR-deficient deletion strain Δ*frdCAB*. B. In the resulting mutant fMFR the *sdhABE* operon is under control of the strong *frd* promoter. This system allows introduction of mutations and affinity tags in *sdhA*. Open reading frames are indicated by grey arrows including locus tags. The ruler gives base pairs with respect to the *Wolinella succinogenes* DSM 1740 complete genome sequence (NC_005090). Correct integration was verified via PCR with the primer pair Cat2_seq1 and wssdhB1R and sequencing of the resulting DNA fragment and Southern blot. C. Southern blot of cut genomic DNA from mutants fMFR and Δ*frdCAB*. The *frdP* probe (clear box) hybridizes with the *frd* promoter and detects the 4307 bp *Eco*RI and the 2948 bp *Hind*III fragment, respectively, in the mutant fMFR. The probe also detects the *frd* promoter at its native locus (second bands in lanes fMFR). An insertion of a 1.3 kb insertion sequence is known to occur in the genome of the deletion mutant upstream of the *kan* gene that replaces the *frdCAB* genes [IS*1302*, see Simon and Kröger (1998) for details]. Consequently both variants with and without insertion sequence were used as positive control (lanes Δ*frd*_*IS*_ and Δ*frd* respectively). IS*1302* also introduces an additional *Eco*RI restriction site leading to a smaller *Eco*RI fragment in Δ*frd*_*IS*_ than in Δ*frd*. Fragment sizes were calculated from a calibration curve with the five marker bands using the program un-scan-it™ (Silk Scientific).

In contrast to all other SQORs characterized in this respect (see Singer and McIntire, 1984; Lancaster, 2004 for reviews), the flavoproteins of the SdhABE complexes from *W. succinogenes* and *C. jejuni* are predicted to harbour non-covalently bound FAD found so far only in soluble fumarate reductases of *Shewanella* species (Bamford *et al*., 1999; Leys *et al*., 1999; Taylor *et al*., 1999). The respective histidine residue required for the covalent linkage is not conserved in these proteins. Experiments on *Saccharomyces cerevisiae* SQR mutants demonstrated that a covalently bound FAD is a requirement for succinate oxidation activity (Robinson *et al*., 1994), a first indication to doubt the assigned succinate dehydrogenase function of the *W. succinogenes* SdhABE complex.

The SdhA of both *W. succinogenes* and *C. jejuni* contains an approximately 40-amino-acid residue extension compared with all other SQORs and harbours a salient twin arginine motif – a signal for the protein export via the tat-pathway (Palmer *et al*., 2005). All other SQORs are oriented towards the cytoplasm (Lancaster, 2002). The iron–sulphur cluster composition in SdhB is characteristic for E-type SQORs; the cysteine pattern implies the presence of a second [4Fe−4S] cluster instead of a [3Fe−4S] cluster, as first discussed for *A. ambivalens* SQR (Gomes *et al*., 1999). The unusual membrane anchor SdhE contains a striking cysteine-rich region referred to as 10-cysteine motif and is phylogenetically related to succinate dehydrogenases and heterodisulphide reductases from a variety of different organisms (Fig. 1) among them not only proteobacteria and archaea but also cyanobacteria and green sulphur bacteria. Furthermore the hydrophobic subunit is predicted to contain no transmembrane domains; membrane association is most likely mediated via amphipathic helices.

Here we present, in the framework of a functional genomics project, the first genetic system for the manipulation and production of a non-classical SQOR. Biochemical characterization of this enzyme reveals that the putative succinate dehydrogenase is in fact a novel 8-methylmenaquinol:fumarate reductase (MFR) with no detectable succinate dehydrogenase activity. Studies on variant enzymes show that the hydrophilic subunits of this complex are, in contrast to all other members of the superfamily, exported into the periplasm via the tat-pathway.

## Results and discussion

### Genetic system, homologous production and purification of the SdhABE complex

Transformation of the *W. succinogenes* QFR deletion mutant Δ*frdCAB* with the vector pSdhA carrying the *sdhA* gene and the *frd* promoter results in the new mutant fMFR (Fig. 2). Integration of the vector at the *sdh* locus puts the *sdhABE* operon under control of the strong *frd* promoter (Fig. 2). The mutant fMFR does not support growth with fumarate as sole electron acceptor; fumarate was complemented with nitrate. In contrast to the deletion mutant, the cell homogenate of the nitrate-grown fMFR strain displays significant fumarate reduction activity (Table 1). Therefore the *sdhABE* operon encodes for a real and active protein which is produced homologously in the *W. succinogenes* mutant fMFR. After membrane preparation from mutant cells, a large proportion of the total activity is found in the soluble fraction, an indication that the SdhABE complex is membrane associated rather than tightly membrane bound. Compared with the *A. ambivalens* enzyme which is clearly a membrane bound protein (Gomes *et al*., 1999), a much larger part of the activity is found in the soluble fraction (Table 1). It can be speculated that the reason for the stronger membrane attachment of the *A. ambivalens* enzyme may be due to the presence of a second hydrophobic subunit (F) (Lemos *et al*., 2001) which is not encoded by the *W. succinogenes* and *C. jejuni* genome. In this work a purification procedure could be established to enrich the protein 24-fold via a combination of anion exchange and gel filtration chromatography with a yield of 36% of the initial activity in the periplasm extract (Table 2). The presence of all three subunits in the preparation could be identified by peptide mass fingerprint analysis (see Fig. S1). Subunits B and C cannot be stained well, as observed previously for the *W. succinogenes* QFR subunits B and C.

**Table 1:** Specific enzymatic activities of the mutant fMFR cell homogenate, soluble fraction and membranes as determined by monitoring the reduction of fumarate by benzylviologen radicals.

	Specific activities (U mg^−1^)
Wild-type cell homogenate (QFR)	1.45
Δ*frdCAB* cell homogenate	< 0.01
Mutant fMFR cell homogenate	0.48
Mutant fMFR soluble fraction	0.20
Mutant fMFR membranes	0.14

As positive and negative control cell homogenate activities of the wild type and the Δ*frdCAB* mutant are shown respectively. One unit (U) of activity is defined as the consumption of 1 μmol fumarate per minute at 37°C.

**Table 2:** Enrichment of the SdhABE complexes MFR, MFR–A86H, MFR–HT and MFR–AH1 and cleavage of the amino-terminal signal peptide.

	Specific activities (U mg^−1^)		Enrichment fold		Yield %		Specific activities (U mg^−1^)	
Purification step	MFR	A86H	MFR	A86H	MFR	A86H	HT	AH1
Periplasm extract	0.7	0.1	1	1	100	100	0.7	0.1
Anion exchange	6.5	0.2	9	3	81	83	6.5	0.8
Gel filtration	16.5	1.1	24	18	36	6		
6xhis-tag positions	No tag						1	37
Western blot signal							(−)	(+)

Only the variant SdhA subunit with a 6xhis-tag on amino acid position 37 (AH1) could be detected on a Western blot treated with an anti-pentahistidine antibody.

To our knowledge, no genetic system for the manipulation and production of a non-classical SQR has been established so far. The system established here allows the study of the putative succinate degydrogenase in the non-pathogenic host *W. succinogenes* with relevance also to the analogous enzyme of the human pathogen *C. jejuni*. The genetic system established in this work allows the introduction of mutations and affinity tags in *sdhA*. In an experiment analogous to the heterologous production of the QFR from *Helicobacter pylori* and *C. jejuni* in *W. succinogenes* (Mileni *et al*., 2006), the initial attempt to clone the whole *sdhABE* operon in the vector pFrdcat2 failed. The *frd* promoter from *W. succinogenes* on the vector pFrdcat2 is also recognized by the *Escherichia coli* strain used for vector amplification (Körtner, 1989), so it can be speculated that the SdhABE complex is produced and toxic in *E. coli*.

### Catalytic properties of the SdhABE complex

**Fig. 3:**
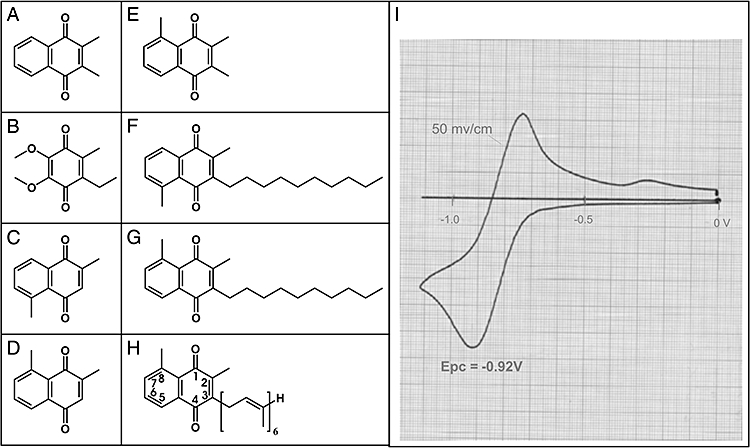
Chemical structures of quinone compounds. See also *Chemical synthesis of quinones*. A. 2,3-Dimethyl-1,4-naphthoquinone (DMN). B. 2,3-Dimethoxy-5-ethyl-6-methyl-1,4-benzoquinone (EQ-0). C. 2,5-Dimethyl-1,4-naphthoquinone. D. 2,8-Dimethyl-1,4-naphthoquinone. E. 2,3,5-Trimethyl-1,4-naphthoquinone (TMN). F. The 5-MMK-6 analogue: 3-decyl-2,5-dimethyl-1,4-naphthoquinone. G. The 8-MMK-6 analogue: 2-decyl-3,5-dimethyl-1,4-naphthoquinone. H. 8-Methylmenaquinone-6 (8-MMK-6). I. Cyclic voltammogram of the quinone compound 3g (8-MMK-6 analogue); *c* = 1 mM; *x* = 100 mV cm^−1^; *i* = 1 mA; *v* = 50 mV s^−1^.

The catalytic properties of the purified enzyme were examined using various possible electron donor and acceptor substrates (Table 3). Strikingly, for the SdhABE complex annotated as a ‘succinate dehydrogenase’ no succinate oxidation activity could be detected. This was independent of whether methylene blue, ferricenium or DCPIP was used as the electron acceptor. One might argue that this could be due to the lack of a suitable binding site for these artificial compounds. However, no succinate oxidation activity could be found using the high-potential quinone EQ-0 (Fig. 3B, Madej *et al*., 2006b) as electron acceptor, although the complex catalyses quinol oxidation and hence harbours a quinone binding site (Table 3). The reason for the lack of succinate oxidation activity could be explained by the absence of a covalently bound FAD which seems to be a prerequisite for succinate oxidation activity (Mewies *et al*., 1998). In variants of *E. coli* QFR (Blaut *et al*., 1989) and *S. cerevisiae* SQR (Robinson *et al*., 1994), the histidine residues which establish the covalent bond to the FAD were replaced by other residues. The variant enzymes were assembled correctly but contained a non-covalently bound FAD and lost the ability to oxidize succinate.

**Table 3:** Catalytic properties of the enriched SdhABE (MFR) complex.

					Specific activities (U mg^−1^)	
Electron donor	*E* (mV)	Electron acceptor	*E* (mV)	Δ*E* (mV)	MFR	QFR
Reduced benzylviologen	−374^a^	Fumarate	25^b^	−399	16.5	59.0
Succinate	25^b^	Methylene blue	11^c^	14	< 0.01	28.8
Succinate	25^b^	Ferricenium	380^d^	−355	< 0.01	14.6
Succinate	25^b^	DCPIP	217^e^	−192	< 0.01	
Succinate	25^b^	DMN	−35^f^	60	< 0.01	
Succinate	25^b^	EQ-0	56^g^	−31	< 0.01	
DMNH_2_	−35^f^	Fumarate	25^b^	−60	0.1	7.4
TMNH_2_	−124^h^	Fumarate	25^b^	−149	0.4	1.0
5-MMKH_2_-6 analogue	−124^h^	Fumarate	25^b^	−149	0.2	
8-MMKH_2_-6 analogue	−124^h^	Fumarate	25^b^	−149	0.9	
CoB-SH/CoM-SH	−143^i^	Fumarate	25^b^	−168	<0.01	

a Wardman (1991).

b Ohnishi *et al*. (2000).

c Clark (1960).

d Lehman *et al*. (1990).

e Hägerhäll (1997).

f *in situ* potential in QFR, Madej *et al*. (2006a).

g Madej *et al*. (2006b), Lancaster *et al*. (2008).

h This work.

i Tietze *et al*. (2003).

Selected corresponding values of purified QFR (Lancaster *et al* 2000; 2001; this work) are included for comparison.

In view of the phylogenetic relationship to heterodisulphide reductases which catalyse the reversible reduction of heterodisulphide (CoM-S-S-CoB) to CoM-SH and CoB-SH (Hedderich *et al*., 2005), the SdhABE complex was tested for CoM-SH/CoB-SH oxidation activity. However, no such activity could be detected (Table 3).

Although the complex catalyses fumarate reduction with the menaquinol-6 (MKH_2_-6) analogue 2,3-dimethyl-1,4-naphthoquinol [DMNH_2_; the quinol form of the quinone shown in Fig. 3A (Lancaster *et al*., 2005)] the activities are very low. In addition to menaquinol-6, membranes of *C. jejuni* (Carlone and Anet, 1983; Collins *et al.*, 1984) and of *W. succinogenes* (Collins and Fernandez, 1984) contain a second quinol, 8-methylmenaquinol-6 (8-MMKH_2_-6) (Thummer *et al*., 2007). So far only MKH_2_-6 has been associated with fumarate respiration. In experiments with *W. succinogenes* respiratory enzymes reconstituted in proteoliposomes, it could be shown that fumarate respiration with formate or hydrogen was catalysed only at high activities when MK-6 was incorporated in the liposomes (Biel *et al*., 2002). However the activities of polysulphide respiration with formate or hydrogen of proteoliposomes containing 8-MMK-6 were more than an order of magnitude greater than of those prepared with MK-6 (Dietrich and Klimmek, 2002). Hence formate dehydrogenase and hydrogenase reduce both MK-6 and 8-MMK-6 whereas the respective terminal reductase preferentially oxidizes one of the two quinols.

The novel enzyme characterized in this work couples the oxidation of both the MKH_2_-6 analogue DMNH_2_ and an 8-MMKH_2_-6 analogue (the quinol form of the quinone shown in Fig. 3G, see also Fig. S2) with the reduction of fumarate (Table 3). However, activities are about one order of magnitude higher when the 8-MMKH_2_-6 analogue is used as electron donor, which correlates also with the approximately 50 mV lower redox mid-point potential of the 8-MMKH_2_-6 analogue (see *Chemical synthesis of quinones* below, and Fig. 3). However, the fumarate reductase activity of the SdhABE complex is only doubled if the methyl group is located at position 5 (5-MMKH_2_-6 analogue) and only four times higher when the symmetrical quinol TMNH_2_ (the quinol forms of the quinones shown in Fig. 3F and E respectively) is used as substrate. In analogy to previous investigations (H.R. Nasiri *et al.*, submitted), we can safely assume that the 5-MMK-6 analogue possesses essentially the same oxidation-reduction mid-point potential as the 8-MMK-6 analogue. Consequently, the increase of activity using the 8-methylmenaquinol-6 analogue as electron donor cannot solely be attributed to its lower mid-point potential compared with that of DMNH_2_. Comparison with the results obtained with purified QFR from *W. succinogenes* (right column of Table 3) also indicated that the observed activities are not simply proportional to the driving force of the respective reaction. Thus, these results indicate that the novel enzyme displays a specificity for 8-methylmenaquinol-6 and therefore should more appropriately be referred to as an 8-methylmenaquinol:fumarate reductase (MFR) in future work.

### Orientation of the SdhABE (MFR) complex: the mutants fMFR–R7/8Q, fMFR–HT and fMFR–AH1

In a strategy analogous to that performed previously to show that *W. succinogenes* hydrogenase is exported into the periplasm via the tat-pathway (Groß*et al*., 1999), the genetic system presented in this work was used to create the mutant fMFR–R7/8Q to examine the orientation of the complex and the recognition of the putative amino-terminal tat-signal sequence (Fig. S3). The replacement of arginines 7 and 8 with glutamines in SdhA disrupts the putative signal peptide. The native QFR is oriented towards the cytoplasm and is used as a control in the experiment. The periplasm of wild-type, mutant fMFR and mutant fMFR–R7/8Q cells was extracted and the spheroblasts were subsequently fractionated in cytoplasm and membranes. The respective fractions were tested for fumarate reductase activity with benzylviologen radicals as electron donor. About 60% of the activity of the unmodified MFR complex was found in the periplasm in contrast to about 4% for the variant fMFR–R7/8Q and the wild-type control (Table 4). Therefore, the results demonstrate the periplasmic orientation of the unmodified MFR complex.

**Table 4:** Comparison of enzyme activities in the cytoplasm, periplasm and membranes of unmodified SdhABE (MFR) complex with the MFR–R7/8Q variant.

	% of total activity		
Strain	Periplasm	Cytoplasm	Membranes
Wild type (QFR)	4.4	0.7	94.9
Mutant fMFR	59.5	0.2	40.3
Mutant fMFR–R7/8Q	3.8	12.5	83.8

Activities were determined by monitoring the oxidation of reduced benzylviologen by fumarate. Wild-type QFR is oriented towards the cytoplasm and is used for comparison.

**Fig. 4:**
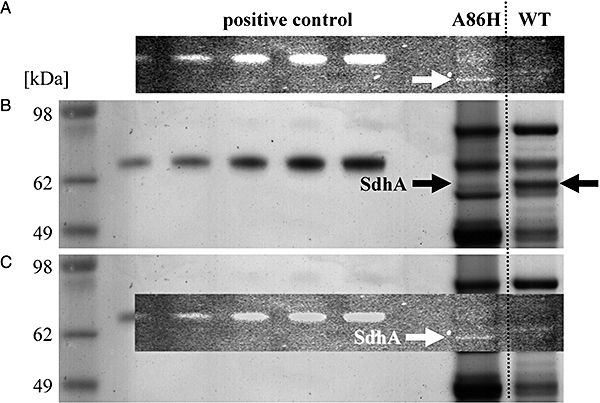
SDS-PAGE of the variant enzyme A86H and the unmodified protein (WT). A. The unstained gel was exposed to UV light; in contrast to the unmodified enzyme a fluorescent band can be detected in the variant A86H. B. The coomassie-stained gel shows the SdhA band for both the variant and the unmodified enzyme. C. Superimposition of the pictures identifies the fluorescent band as SdhA. In the variant enzyme A86H the FAD is covalently bound to the protein.

According to previous studies (Berks *et al*., 2005), the tat signal peptide is cleaved during export at a peptidase recognition sequence corresponding to amino acids 31–33 in SdhA (Fig. 4). The genetic system established in this work allowed us to create variant SdhABE (MFR) complexes containing a 6xhis-tag at amino acid positions 1 and 37 in the mutants fMFR–HT and fMFR–AH1 respectively. The active variant proteins were enriched by anion exchange chromatography and analysed on a Western blot treated with anti-polyhistidine antibodies (Fig. S4). Only the SdhA variant with the his-tag on amino acid position 37 is detectable (Table 2); the amino-terminal signal peptide is most likely cleaved during export.

### Attachment of the FAD: the mutant fMFR–A86H

The amino acid sequence alignment of SdhA with other SQORs (Fig. S5) indicates a non-covalent attachment of the FAD to the protein, the respective histidine is not conserved in the MFR and corresponds to alanine 86. To elucidate the requirements for the covalent attachment of the FAD to a protein the mutant fMFR–A86H was created which encodes for a histidine on position 86 instead of an alanine in SdhA. The active variant enzyme could be enriched (Table 2) and analysed via SDS-PAGE (Fig. 4). In contrast to the unmodified enzyme a fluorescent band corresponding to SdhA could be detected in the variant. The exchange of alanine 86 to histidine is the single additional requirement for the covalent attachment of the FAD to the protein.

In the *W. succinogenes* QFR the FAD is covalently bound to histidine 43 of subunit A (Kenney and Kröger, 1977; Lancaster *et al*., 1999). Amino acids close to the FAD binding site are highly conserved (Fig. S5), mutations in this area in the SQR from *Bacillus subtilis* resulted in the loss of the flavin and enzyme activity (Maguire *et al*., 1986). Both in the SQR from *S. cerevisiae* (Robinson and Lemire, 1996) and in the *E. coli* QFR (Blaut *et al*., 1989; Ackrell *et al*., 1992) the exchange of the FAD-binding histidine resulted in a non-covalently bound FAD, and the enzyme complex remained intact and active. Interestingly these complexes lost their ability to oxidize succinate; the covalent FAD is most likely a prerequisite for succinate oxidation. The observation that the MFR is also unable to catalyse succinate oxidation in detectable amounts supports this hypothesis. Interestingly the variant complex MFR–A86H is still not able to catalyse succinate oxidation with either methylene blue, ferricenium, DCPIP, DMN or EQ-0 as electron acceptors so the covalent linkage alone is not sufficient to support succinate oxidation activity.

Apart from the SdhABE complexes from *C. jejuni* and *W. succinogenes* the FAD-binding histidine is conserved among all SQORs but not in the periplasmic fumarate reductase of *Shewanella* species (Pealing *et al*., 1992). However these enzymes harbour neither iron–sulphur clusters nor a 10-cystein motif but haem groups which distinguishes them from the MFR (Bamford *et al*., 1999; Leys *et al*., 1999; Taylor *et al*., 1999).

### Physiological role

Although no growth conditions have been identified so far under which the *W. succinogenes* MFR complex is produced, it is known that the expression of the *sdhABE* genes encoding for the highly similar putative ‘succinate dehydrogenase’ from the human pathogen *C. jejuni* are regulated in response to growth conditions. The genes are upregulated *in vivo* in the chicken caecum relative to the expression of laboratory-grown bacteria (Woodall *et al*., 2005). It can be reasoned that in the caecum, *C. jejuni* grows under oxygen-limited conditions in contrast to the microaerophilic environment in the laboratory-grown strain. In contrast to findings for *E. coli*, where succinate dehydrogenase is upregulated under aerobic conditions and fumarate reductase in anaerobiosis (Park *et al*., 1995), both the *sdhABE* genes and the fumarate reductase genes are upregulated in *C. jejuni* in an oxygen-limited environment (Woodall *et al*., 2005). Our results reconcile this initially puzzling observation, if we conclude that the *C. jejuni* putative succinate dehydrogenase is in fact a methylmenaquinol:fumarate reductase with properties similar to those of the *W. succinogenes* MFR.

The novel methylmenaquinol:fumarate reductase could be part of an alternative metabolic pathway using the low-potential methylmenaquinol-6 reduced by hydrogenase or formate dehydrogenase. Due to the periplasmic orientation the enzyme could use fumarate directly without the need of importing it into the cytoplasm via the *dcu* dicarboxylate transporters (Janausch *et al*., 2002).

An unsolved question is why the *Wolinella* fMFR mutant is unable to grow by fumarate respiration, although the overproduced MFR enzyme has substantial 8-methylmenaquinol:fumarate reductase activity. Further work is required to examine the role of this enzyme at a physiological level – its properties can provide hints for the screening of new growth conditions in the laboratory. Quantitative RT-PCR of *W. succinogenes* grown under different conditions could clarify some points discussed here. In conclusion, this work is an important step towards the mechanistic understanding of this type of SQORs which are so far not well characterized.

## Experimental procedures

### Bacterial growth

*Wolinella succinogenes* was grown with formate as electron donor and fumarate or nitrate as electron acceptor (Bronder *et al*., 1982; Lorenzen *et al*., 1993). The medium was supplemented with Brain-Heart-Infusion (0.5% w/v; Gibco BRL)

### Plasmid construction

Due to its central significance for the *W. succinogenes* expression system, the sequence of the plasmid pFrdcat2 (Simon *et al*., 1998) was determined in this work and deposited in the EMBL nucleotide sequence database with the Accession No. AM909725.

#### pSdhA

DNA fragments corresponding to the region, which encodes for the SdhA subunit (1850 bp) and the pFrdcat2 vector fragment without *frdCAB* genes (5283 bp), were synthesized by PCR using primers carrying suitable restriction sites for cloning at their 5′ ends. Genomic DNA from *W. succinogenes* with primer pair Sac_Ws-Sdh_Fw and Not_Ws-SdhA_Rv and plasmid pFrdcat2 with primer pair Not_cat2_Fw and Sac_cat2_Rv were used respectively (Table S1). The *sdhA* gene was cloned in the plasmid fragment using *Sac*II and *Not*I restriction (Fig. 2A). The entire *sdhA* gene of the resulting plasmid was sequenced.

#### pSdhAHT

The oligonucleotides SacIIHis6TEV1 and SacIIHis6TEV2 (Table S1) were hybridized by heating them for 10 min at 95°C and slowly cooled to 4°C at a rate of 0.1°C min^−1^. The double-stranded oligonucleotide was inserted at the start of the *sdhA* gene in pSdhA using *Sac*II restriction. The orientation of the oligonucleotide was verified by sequencing.

#### pSdhAH1

To insert a 6xhis-tag at amino acid position 37 in SdhA a PCR fragment was synthesized using suitable primers carrying an *Apa*I restriction site and one of them the bases encoding for the 6 histidines as overhang on their 5′ ends. The vector pSdhA was used as template with the primer pair SdhH1_Fw and SdhH1_Rv (Table S1). The resulting linear PCR fragment was circularized using *Apa*I restriction.

## Site-directed mutagenesis

Site-directed mutagenesis was performed using the QuikChange® site-directed mutagenesis kit from Stratagene and a modified protocol as described in Wang and Malcolm (1999). The vectors pR7/8Q and pA86H were synthesized with the plasmid pSdhA as template and specifically synthesized oligonucleotides carrying the desired nucleotide mismatches (Table S1). The sequence of the respective modified region of the resulting plasmid was determined.

## Transformation of *W. succinogenes*

The transformation of the fumarate reductase activity-deficient deletion mutant Δ*frdCAB* with the constructed plasmids was performed as described in Simon *et al*. (1998). Integration of the plasmid occurs at the native *sdh* locus in the *W. succinogenes* genome and was verified via PCR, Southern blot and sequencing (Fig. 2 and Fig. S4).

## Computer analysis

Database searches and phylogenetical tree representation of resulting data made use of the program blast (Altschul *et al*., 1997). Plasmid/primer design and multiple sequence alignments were performed with Clone Manager 9 (SciEd).

## Chemical synthesis of quinones

### General remarks

NMR spectra were recorded on a Bruker spectrometer AM250 operating at a ^1^H frequency of 250 MHz and ^13^C frequency of 62.9 MHz. Mass spectra (MALDI) were recorded on a Fisons VG TofSpec spectrometer. All reactions were monitored by thin-layer chromatography (TLC), performed on silica gel POLYgram® (Macherey-Nagel). Chromatographic purifications were done with Merck silica gel 60.

### Chemistry

2,3-Dimethyl-1,4-naphthoquinone (DMN; Fig. 3A; Lancaster *et al*., 2005) and 2,3-dimethoxy-5-ethyl-6-methyl-1,4-benzoquinone (EQ-0; Fig. 3B; Madej *et al*., 2006b) were synthesized previously. 2,5-Dimethyl-1,4-naphthoquinone (Fig. 3C), 2,8-dimethyl-1,4-naphthoquinone (Fig. 3D) and 2,3,5**-**trimethyl-1,4-naphthoquinone (TMN; Fig. 3E) were prepared starting from commercial naphthalene 1,6-dimethylnaphthalene, 1,7-dimethylnaphthalene and 1,6,7-trimethylnaphthalene via chromium trioxide oxidation (Schmid and Labahn, 1998). The 5-MMK-6 analogue 3-decyl-2,5-dimethyl-1,4-naphthoquinone (Fig. 3F) and the 8-MMK-6 analogue 2-decyl-3,5-dimethyl-1,4-naphthoquinone (Fig. 3G) were synthesized starting from 2,5-dimethyl-1,4-naphthoquinone (Fig. 3C) and 2,8-dimethyl-1,4-naphthoquinone (Fig. 3D) respectively. The formal introduction of the alkyl side-chain was achieved by radical alkylation via a Hunsdiecker oxidative decarboxylation of undecanoic acid, promoted by silver nitrate as a catalyst in the presence of ammonium peroxydisulphate in a acetonitrile/water mixture as previously described (Madej *et al*., 2006a). The purification of the crude products by silica chromatography (hexane : ethylacetate 6:1) yielded 8-MMK-6 analogue (3g) and 5-MMK-6 analogue (3c) at 44.2% and 48% yields respectively.

### Electrochemistry

Cyclovoltammetry experiment (Fig. 3I) was measured using a conventional three-electrode cell in dimethoxyethane with tetrabutylammoniumperchlorate as supporting electrolyte on platinum working and counter electrodes. The concentration of 2-decyl-3,5-dimethyl-1,4-naphthoquinone (Fig. 3G) was 1 mM. The potential is given versus Ag/AgCl reference electrode, with a voltage sweep rate of 50 mV s^−1^. 1,2-Dimethoxyethane (DME) was purchased from Fluka, puriss; dried over molecular sieves (H_2_O < 0.005%). Solution of 2-decyl-3,5-dimethyl-1,4-naphthoquinone was deoxygenated with dry nitrogen and maintained under an inert nitrogen atmosphere at 25°C (H.R. Nasiri *et al.*, submitted).

### 2,5-Dimethyl-1,4-naphthoquinone *(Fig. 3C)*

^1^H-NMR (250.13 MHz, CDCl_3_) as previously reported (Schmid and Labahn, 1998); ^13^C-NMR (62.9 MHz, CDCl_3_): *δ* (p.p.m.) = 187.0; 185.9 (C=O), 146.2; 140.9; 133.6; 129.7 (C), 137.8; 137.4; 132.6; 125.4 (CH), 22.5; 15.9 (CH_3_).

### 2,8-Dimethyl-1,4-naphthoquinone *(Fig. 3D)*

^1^H-NMR (250.13 MHz, CDCl_3_) as previously reported (Schmid and Labahn, 1998); ^13^C-NMR (62.9 MHz, CDCl_3_): *δ* (p.p.m.) = 187.4; 185.3 (C=O), 149.4; 141.2; 133.7; 129.7 (C), 137.8; 134.2; 132.7; 124.9 (CH), 22.8; 16.8 (CH_3_).

### 2,3,5-Trimethyl-1,4-naphthoquinone *(Fig. 3E)*

^1^H-NMR (250.13 MHz, CDCl_3_) as previously reported (Schmid and Labahn, 1998); ^13^C-NMR (62.9 MHz, CDCl_3_): *δ* (p.p.m.) = 186.9; 185.2 (C=O), 144.1; 141.7; 140.7; 133.6; 129.9 (C), 137.2; 132.3; 125.1 (CH), 22.7; 13.0; 12.5 (CH_3_).

### The 5-MMK-6 analogue 3-decyl-2,5-dimethyl-1,4-naphthoquinone *(Fig. 3F)*

^1^H-NMR (250.13 MHz, CDCl_3_): *δ* (p.p.m.) = 8.00 (m, 1H, aromatic proton), 7.60 (m, 2H, aromatic protons), 2.98 (s, 3H, CH_3_), 2.70 (m, 2H, *CH*_*2*_CH_2_), 2.1 (s, 3H, CH_3_) 1.1–1.7 (m, 16H, 8xCH_2_), 0.89 (s, 3H, CH_3_). ^13^C-NMR (62.9 MHz, CDCl_3_): *δ* (p.p.m.) = 186.7; 185.8 (C=O), 148.8; 141.3; 140.7; 133.6; 129.9 (C), 137.3, 132.3, 125.0 (CH), 31.9; 30.1; 29.5; 29.4; 29.3; 28.9; 27.3; 22.9; 22.8 (CH_2_), 22.6; 14.1; 12.4 (CH_3_).

### The 8-MMK-6 analogue 2-decyl-3,5-dimethyl-1,4-naphthoquinone *(Fig. 3G)*

^1^H-NMR (250.13 MHz, CDCl_3_): *δ* (p.p.m.) = 7.90 (d, *J* = 7.5 Hz, 1H, aromatic proton), 7.45 (m, 2H, aromatic protons), 2.67 (s, 3H, CH_3_), 2.39 (t, *J* = 7.3 Hz 2H, *CH*_*2*_CH_2_), 2.14 (s, 3H, CH_3_) 1.1–1.6 (m, 16H, 8xCH_2_), 0.83 (s, 3H, CH_3_). ^13^C-NMR (62.9 MHz, CDCl_3_): *δ* (p.p.m.) = 187.4; 185.1 (C=O), 145.8; 144.8; 140.6; 133.6; 125.1 (C), 137.2, 133.8, 125.1 (CH), 31.8; 29.9; 29.5; 29.5; 29.4; 29.2; 28.6; 26.8; 22.8 (CH_2_), 22.6; 14.1; 12.8 (CH_3_). MS (MALDI): 326.48 (M).

E_0_ = −920 mV, 50 mV lower than that for DMN/DMNH_2_ (Madej *et al*., 2006a).

## Preparation of the SdhABE (MFR) complex

Frozen *W. succinogenes* cells were three times thawed and frozen in liquid nitrogen and suspended in 2 ml g^−1^ wet cells anoxic buffer containing 50 mM TRIS·Cl (pH 8.25), 1 mM malonate and dithiothreitol (DTT) respectively. The suspension was centrifuged for 15 min at 20 000 *g* and 0.1% w/v laurylmaltoside (LM) was added to the supernatant and all buffers. After stirring for 30 min at room temperature the mixture was centrifuged in an ultracentrifuge for 45 min at 190 000 *g*. The supernatant was applied on a HiPrep™ 16/10 DEAE Sepharose™ column using an ÄKTApurifier™ system (GE Healthcare) and eluted with 0.15 M NaCl. The eluated fractions were tested for fumarate reductase activity using benzylviologen radicals as electron donor. Fractions containing at least 10% of the maximum activity were pooled and concentrated in a pressure dialysis cell (50 kDa cut-off) and further purified via gel filtration using a HiLoad™ 16/60 Superdex™ 200 column on the ÄKTApurifier™ system with anoxic buffer containing 50 mM TRIS·Cl (pH 8.25), 0.2 M NaCl, 1 mM malonate, 1 mM DTT and 0.1% LM.

## Enzymatic activities

All enzymatic assays were performed at 37°C in 0.4 cm path-length anoxic quartz cuvettes.

Fumarate reduction activity was measured in anoxic buffer containing 50 mM TRIS·Cl (pH 8.0) by monitoring photometrically the oxidation of dithionite-reduced benzylviologen (BV, ε_546_ 19.5 mM^−1^ cm^−1^) by fumarate as described (Unden and Kröger, 1986). The 1 ml assay mixture contained 1 mM benzylviologen titrated with sodium dithionite (20 mg ml^−1^) to an optical density at 546 nm of 2 and 10 μg of enriched MFR solution; the reaction was started by the addition of 10 mM fumarate. The SdhABE (MFR) complex was tested for succinate oxidation activity using either methylene blue (MB, ε_578_ 17.1 mM^−1^ cm^−1^) (Kröger *et al*., 1980), dichlorophenolindophenol (DCPIP, ε_578_ 15.3 mM^−1^ cm^−1^) (Lemma *et al*., 1990), ferricenium hexafluorophosphate (Lehman *et al*., 1990), dimethylnaphthoquinone (DMN) (Unden and Kröger, 1981) or EQ-0 (Madej *et al*., 2006b) as electron acceptor. The 1 ml assay mixture contained 10–80 μg of enriched MFR solution as well as 0.2 mM methylene blue, DCPIP, ferricenium, DMN or EQ-0 respectively. The reaction was started by the addition of 10 mM succinate. The reduction of fumarate with CoM-S-H and Co-B-S-H was tested as described (Heim *et al*., 1998). The reaction mixture contained 10 μg of enriched MFR solution and 4 mM of both CoM-S-H and Co-B-S-H. The reaction was started by the addition of 10 mM fumarate. The oxidation of the menaquinol analogue DMNH_2_ (ε_270−290_ 15.2 mM^−1^ cm^−1^) with fumarate was determined as described (Unden and Kröger, 1981). The 1 ml assay mixture contained 0.2 mM DMNH_2_ reduced with about 10 μl of NaBH_4_ (5 mg ml^−1^) and 20 μg of enriched MFR solution. The reaction was started by the addition of 1 mM fumarate.

The synthesized quinones (TMN, the 5-MMK-6 analogue and the 8-MMK-6 analogue) were reduced with dithionite according to Rich (1978). The quinones were put in an anoxic vial and dissolved in diethylether (5 mM). An equal volume of aqueous sodium dithionite solution (20 mg ml^−1^) was added and the whole was vigorously shaken. The quinol layer was decanted and transferred to a fresh anoxic vial using a gas-tight syringe. The ether was evaporated under vacuum and the vial was flushed with argon. The quinol was then dissolved in anoxic ethanol. The quinol oxidation activity was measured in 50 mM sodium phosphate buffer (pH 7.4) by monitoring the change of the absorbance difference between 270 and 290 nm. The 1 ml reaction mixture contained 20 μg of enriched MFR solution and 0.2 mM TMN, 0.06 mM 5-MMK-6 analogue or 0.06 mM 8-MMK-6 analogue respectively. The reaction was started by the addition of 1 mM fumarate. The molar extinction coefficient of the 8-MMK-6 analogue was calculated from air-oxidized and NaBH_4_-reduced spectra: ε_270−290_ 10.5 ± 1.5 mM^−1^ cm^−1^ (Fig. S2). The same value was used to calculate the activity with the TMN and the 5-MMK-6 analogue.

## Membrane preparation

Frozen cells were suspended with anoxic buffer containing 50 mM TRIS·Cl (pH 7.8), 2 mM malonate, 1 mM DTT and disrupted in a French Pressure Cell (130 MPa; 10 ml min^−1^ flow). Membranes were prepared from the cell homogenate via ultracentrifugation (200 000 *g*, 60 min).

## Separation of the periplasm, cytoplasm and membranes

*Wolinella succinogenes* mutant fMFR cells were harvested in the exponential growth phase, suspended in cold anoxic buffer containing 50 mM TRIS·Cl (pH 8.0), 0.5 M mannitol, 10 mM EDTA, 0.5 mg ml^−1^ lysozyme and gently mixed with a vortexer for 1 min. The mixture was subsequently centrifuged (14 000 *g*, 1.5 min; 4°C) to separate the periplasm extract (supernatant) from the spheroblasts which were suspended in cold anoxic buffer containing 10 mM TRIS·Cl (pH 8.0), 15 mM magnesium chloride and 1.5 g l^−1^ DNase I. To lyse the spheroblasts and separate the membranes from the cytoplasm the mixture was gently mixed with a vortexer for 5 min and centrifugated (20 000 *g*, 15 min, 4°C) (Krafft *et al*., 1995).

## Protein characterization

SDS-PAGE was performed according to Laemmli (1970) using 4–12% polyacrylamide gradient gels (Invitrogen) and Coomassie Blue for staining. Detection of covalently bound FAD was carried out on a SDS-PAGE gel washed for 10 min in 10% acetic acid and irradiated with UV light (*λ* = 302 nm) before staining. The total protein concentration was measured with the BCA (bicinchoninic acid) assay (Pierce Biotechnology). For Western blot analysis, protein was transferred from the gel to nitrocellulose by electroblotting in a discontinuous buffer system (Kyhse-Andersen, 1984). The anti-pentahistidine-alkaline phosphatase antibody (Sigma) was applied at a dilution of 1:2000.

## Peptide mass fingerprint analysis

The mass-spectrometric protein identification was performed as described (Rais, 2005). The respective protein bands were cut from an coomassie-stained SDS-PAGE gel and destained with a 100 mM ammoniumhydrogencarbonate (AHC)/50% acetonitrile (ACN) mixture before shrinking the gel pieces with pure ACN. The dried pieces were soaked with trypsin solution (20 μg ml^−1^, Promega), covered with 40 mM AHC and incubated overnight at 37°C. Extraction of the cut peptides was performed via ultrasonification and addition of 2 μl of 10% trifluor acetic acid (TFA) to the supernatant. The solution was evaporated in a vacuum centrifuge and the pellet dissolved in 70% ACN/0.1% TFA. A saturated solution of α-cyano-4-hydroxy-cinnamic acid in 70% ACN/0.1% TFA was prepared. Equal parts of the peptide solution and a suitable matrix dilution were applied to the target, dried and washed with ice-cold formic acid. The peptide fragments were examined using in a ‘Omni Flex®’ MALDI-TOF spectrometer (Bruker). Resulting peaklists (Fig. S1) were compared with the MSDB database using the program ‘Mascot’ (Matrix Science).
