# Supplementary material for: Production, characterization and determination of the real catalytic properties of the putative ‘succinate dehydrogenase’ from Wolinella succinogenes
Source: Mol Microbiol. 2009 Jan 16;71(5):1088–101. doi: 10.1111/j.1365-2958.2008.06581.x (PMC2680327; doi:10.1111/j.1365-2958.2008.06581.x)
Supplement: Supplementary file 1 [file mmi0071-1088-SD1.pdf]

Supporting Information:

Production, Characterization, and Determination of the Real Catalytic Properties of the Putative 'Succinate Dehydrogenase' from *Wolinella succinogenes*

Hanno D. Juhnke[1,||], *Heiko Hiltscher*[1,†], *Hamid R. Nasiri*[2,‡], *Harald Schwalbe*[2], *and C. Roy D. Lancaster* [1,3,*]

[1] Cluster of Excellence "Macromolecular Complexes", Max Planck Institute of Biophysics, Dept. of Molecular Membrane Biology, Max-von-Laue-Str. 3, D-60438 Frankfurt am Main, Germany
[2] Cluster of Excellence "Macromolecular Complexes", Institute of Organic Chemistry and Chemical Biology, Center for Biomolecular Magnetic Resonance, Johann Wolfgang Goethe University, Max-von-Laue-Str. 7, D-60438 Frankfurt am Main, Germany
[3] Department of Structural Biology, Faculty of Medicine, Saarland University, D-66421 Homburg (Saar), Germany
[||] present address: Sanofi-Aventis Deutschland GmbH, QA Operations, Industriepark Höchst, Bldg. H500, D-65926 Frankfurt am Main, Germany
† present address: Botanisches Institut, Heinrich-Heine-Universität Düsseldorf, Universitätsstr. 1, D-40225 Düsseldorf, Germany
[‡] present address: Department of Chemistry, University of Cambridge, Lensfield Road, Cambridge CB2 1EW, United Kingdom
* To whom correspondence should be addressed: Dept. of Structural Biology, Faculty of Medicine, Saarland University, D-66421 Homburg (Saar), Germany,
E-mail: Roy.Lancaster@structural-biology.eu, Tel.: +49 6841 1626 235, Fax.: +49 1212 616 041 965

SUPPORTING TABLE 1. Strains, plasmids and oligonucleotide primers used in the present study.

SUPPORTING FIGURE 1. SDS-PAGE for peptide mass fingerprint analysis.

SUPPORTING FIGURE 2. Determination of the molar oxidized-minus-reduced absorption difference coefficient at 270 nm of the 8-MMKH$_2$-6 analog 2,8-dimethyl-3-decyl-1,4-dihydroxynaphthoquinol.

SUPPORTING FIGURE 3. Sequence alignment of the amino-termini of *W. succinogenes* (*Ws*), *C. jejuni* (*Cj*), *A. ambivalens* (*Aa*) and *Synechocystis* (*Sc*) SdhA.

SUPPORTING FIGURE 4. Western blot analysis of the variant SdhABE complexes MFR-HT and MFR-AH1.

SUPPORTING FIGURE 5. Comparison of residues involved in fumarate reduction in the *W. succinogenes* quinol:fumarate reductase and the MFR.

SUPPORTING REFERENCES

SUPPORTING TABLE 1.

Strains, plasmids and oligonucleotide primers used in the present study.

Strain, plasmid or primer	Relevant properties, usage, nucleotide sequence	Reference
Bacterial strains		
W. succinogenes DSMZ 1740	Wild-type strain	(Simon *et al.*, 2000)
W. succinogenes Δ*frdCAB*	Fumarate reductase operon (*frdCAB*) deletion mutant	(Simon *et al.*, 1998)
W. succinogenes fMFR	Strain expressing the *sdhABE* operon under control of the *frd* promoter	This study
W. succinogenes fMFR-R7/8Q	=fMFR; arginines 7 and 8 in sdhA are replaced by glutamines	This study
W. succinogenes fMFR-A86H	=fMFR; alanine 86 replaced by a histidine	This study
W. succinogenes fMFR-HT	=fMFR; amino-terminal (*sdhA*) His6-tag plus TEV protease site	This study
W. succinogenes fMFR-AH1	=fMFR; His6-tag introduced at amino acid position 37	This study
E. coli XL1-blue	Strain used for cloning and plasmid propagation	Stratagene
Plasmids		
pFrdcat2	Derivative of pSC101; *E. coli* low-copy vector containing the *W.* succinogenes *frdCAB* genes including *frd* promoter and terminator sequences	(Simon *et al.*, 1998); sequence determined in this study
pSdhA	Derivative of pFrdcat2 containing the *sdhA* gene instead of *frdCAB* genes from *W. succinogenes*	This study
pR7/8Q	Derivative of pSdhA; codons for arginines 7 and 8 are replaced by codons for glutamines	This study
pA86H	Derivative of pSdhA; codon for alanine 86 is replaced by a codon for histidine	This study
pSdhHT	Derivative of pSdhA; codons encoding for six histidines plus TEV protease site are inserted in *sdhA*	This study
pSdhAH1	Derivative of pSdhA; codons encoding for six histidines at amino acid position 37 are inserted in *sdhA*	This study
Primers		
Not_cat2_Fw	TATCAAATGCGGCCGCATCTCCTTGGAGCGGGGTCTCC Forward primer used for the amplification of the pFrdcat2 vector fragment; contains a *Not*I restriction site (underlined)	This study
Sac_cat2_Rv	TCCCCGCGG**CAT**CTGTTTCCCCTGTGCAGTATTG Reverse primer used for the amplification of the pFrdcat2 vector fragment; contains a *Sac*II restriction site (underlined) and the *frdC* start codon (in boldface)	This study
Sac_Ws-Sdh_Fw	TCCCCGCGGAGTGAACAATTTACCCGAAGGGAG Forward primer	This study

	used for the amplification of *sdhA* from *W. succinogenes* genomic DNA; contains a *Sac*II restriction site (underlined)	
Not_Ws-SdhA_Rv	TATCAAATGCGGCCGCTTAGTATTTCCTCTCCTCAATTTTGAATTG Reverse primer used for the amplification of *sdhA* from *W. succinogenes* genomic DNA; contains a *Not*I restriction site (underlined)	This study
cat2_seq1	CTCTTACAGTTCCAAACTACC binds in the *frd* promoter on the plasmid pSdhA; a PCR product with primer wssdhB1R indicates correct integration of pSdhA in the Δ*frdCAB* genome	This study
wssdhB1R	GAGCGTGATGTCTTGGGTTTGC binds in *sdhB* on the Δ*frdCAB* genome	This study
SdhR7/8Q1	CGCGGAGTGAACAATTTACCCAACAGGAGTTTCTTCAGTCTGCC	This study
SdhR7/8Q2	GGCAGACTGAAGAAACTCCTGTTGGGTAAATTGTTCACTCCGCG Complementary mutagenic primers used for the replacement of arginines 7 and 8 to in SdhA to glutamines via site-directed mutagenesis; the changed codons are underlined	
A86H1	GGTTATGCCCACCCGAAGCCACACCACAATGGCTGAAGG	The present study
A86H2	CCTTCAGCCATTGTGGTGTGGCTTCGGGTGGGCATAACC Complementary mutagenic primers used for the replacement of alanine 86 to histidine in SdhA via site-directed mutagenesis; the changed codon is underlined	
SacIIHis6TEV1	TCCCCGCGG**CACCATCACCATCACCAT**GAGAATCTCTATTTTCA AGGACCGCGGCCT Encodes for a His6-tag (in boldface) plus TEV protease site; used for insertion in the *Sac*II restriction site of pSdhA; contains two *Sac*II restriction sites (underlined)	This study
SacIIHis6TEV2	AGGCCGCGGTCCTTGAAAATAGAGATTCTC**ATGGTGATGGTGAT GGTG**CCGCGGGGA Complementary to SacIIHis6TEV1	This study
SdhH1_Fw	ATGGGCCCCTCCCCATCAATACCTCTGGCATCCCC Forward primer used for the insertion of a His6-tag at amino acid position 37 via PCR from pSdhA; contains a *Apa*I restriction site (underlined)	This study
SdhH1_Rv	TAGGGCCCGGC**ATGGTGATGGTGATGGTG**GGAAGAGGAGGCAA AAGCCCTATCAACCCC Reverse primer used for the insertion of a His6-tag at amino acid position 37 via PCR from pSdhA; contains a *Apa*I restriction site (underlined) and codons for 6 histidines (in boldface)	This study

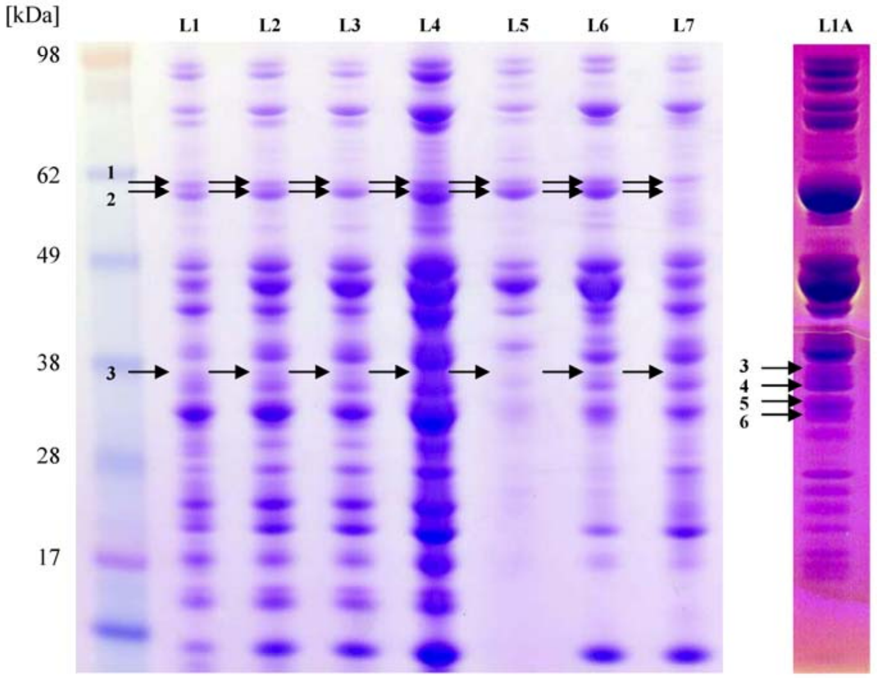

b nr.	s	m [kDa]	pI	em	fm	sc	protein
**1**	78	66.5	7.29	24	12	28%	***´Succinate Dehydrogenase´ Flavoprotein Subunit, W. succinogenes***
**2**	97	60	6.15	49	21	34%	***Dihydroxy-Acid Dehydratase, W. succinogenes***
**3**	76	35.7	7.53	52	11	37%	***´Succinate Dehydrogenase´ Iron-Sulfur Protein, W. succinogenes***
**4**	65	31.8	7.82	16	8	33%	***Co-Chaperone-Curved DNA Binding Protein A (CBPA), W. succinogenes***
**5**	55	29.2	5.65	25	7	41%	***Septum Site-Determining Protein Mind Cell Division Inhibitor, W. succinogenes***
**6**	111	30.7	8.21	25	11	36%	***´Succinate Dehydrogenase´ Subunit C, W. succinogenes***

SUPPORTING FIGURE 1. SDS-PAGE for peptide mass fingerprint analysis. Lanes 1-3: fractions with highest activity after 1st ion exchange chromatography, 20 µg protein per lane; lane 4: pooled and concentrated fractions after 1st ion exchange chromatography ,30 µg; lanes 5 and 6: fractions of highest activity after gel filtration, 5 µg; lane 7: pooled and concentrated fractions after gel filtration, 5 µg; lane 1A: fraction of highest activity after gel filtration, different preparation. The two prominent bands 1 and 2 and all bands between 38 kDa and 28 kDa were cut out, treated with trypsin and examined via MALDI-TOF. Assignment of the peaklists to the respective proteins was performed with the program ‘Mascot’. Only assignments with a score of 54 and higher are shown (bands indicated by an arrow). Abbreviations: b nr.: band number; s: score; m: calculated mass from protein sequence; pI:

theoretical isoelectric point; em: entered mass values; fm: fitting mass values; sc: sequence coverage; protein: database output.

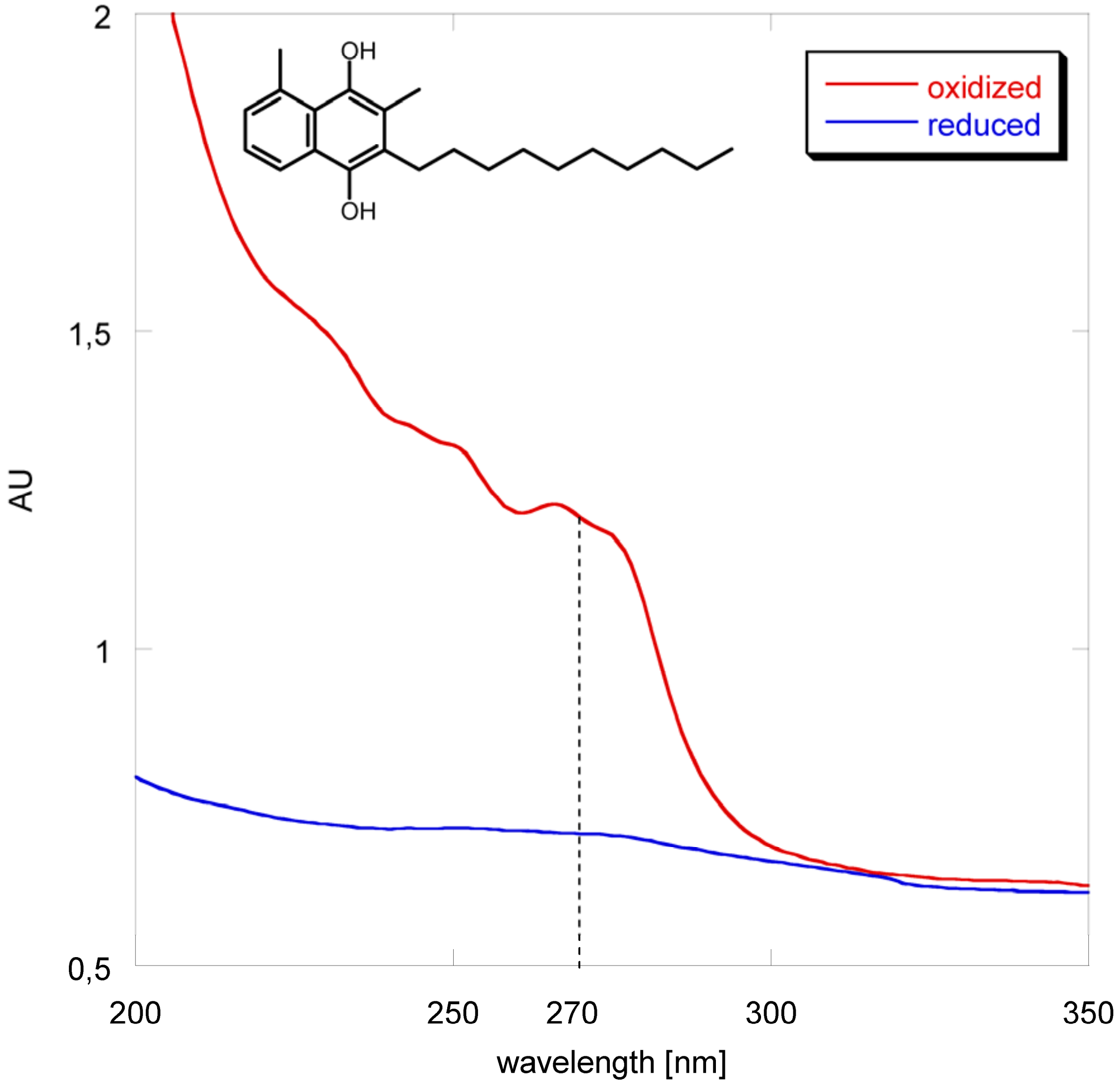

SUPPORTING FIGURE 2. Determination of the molar oxidized-minus-reduced absorption difference coefficient at 270 nm of the 8-methylmenaquinol-6 analog 2,8-dimethyl-3-decyl-1,4-dihydroxynaphthoquinol (the quinol form of the quinone shown in Fig 3g). The coefficient was determined from air-oxidized quinone and KBH_4-reduced quinol at a concentration of 0.1 µM.

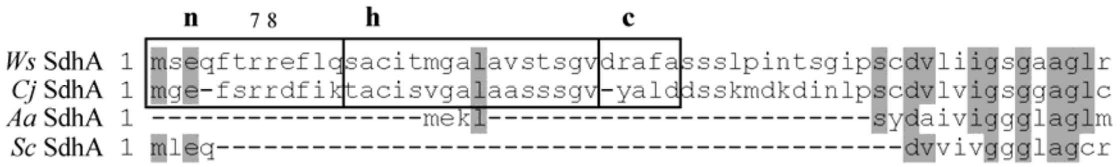

SUPPORTING FIGURE 3. Sequence alignment of the amino-termini of *W. succinogenes* (*Ws*), *C. jejuni* (*Cj*), *A. ambivalens* (*Aa*) and *Synechocystis* (*Sc*) SdhA. The amino-termini from the *W. succinogenes* and *C. jejuni* gene product are significantly longer than in the *A. ambivalens* and *Synechocystis* enzyme and harbour a twin-arginine motif (consensus sequence s/t-r-r-e-f-l-k/q), a signal for the export into the periplasm via the twin-arginine translocase (Tat) pathway. The amino acid composition corresponds to the generic tripartite structure (n-, h-, and c-region) of twin-arginine signal peptides (Palmer *et al.*, 2005). This signal is absent in the sequences of *A. ambivalens* and *Synechocystis* SdhA.

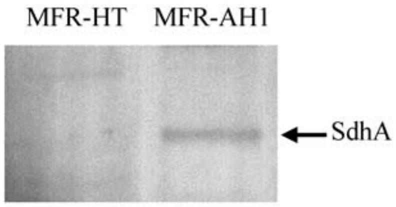

SUPPORTING FIGURE 4. Western blot analysis of the variant SdhABE complexes MFR-HT and MFR-AH1. The variant enzymes carry a 6xhis-tag on amino acid position 1 and 37, respectively (pooled fractions after anion exchange). However on a Western blot treated with anti-polyhistidine antibodies only the tag on amino acid position 37 is detectable.

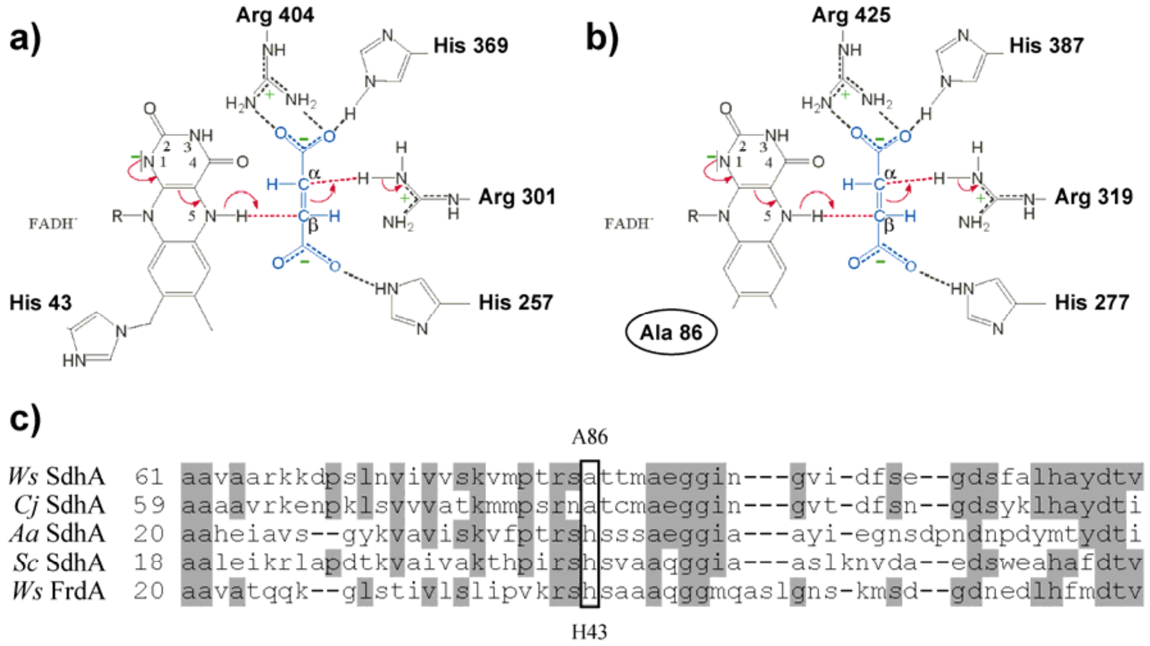

SUPPORTING FIGURE 5. Comparison of residues involved in fumarate reduction in the *W. succinogenes* quinol:fumarate reductase a) and the MFR b) (figure modified from Lancaster *et al.,* 2001). All residues required for the proposed mechanism are conserved except for the FAD-binding histidine 43 which corresponds to alanine 86 in the MFR. c) Sequence aligment of *W. succinogenes* (*Ws*), *C. jejuni* (*Cj*), *A. ambivalens* (*Aa*), *Synechocystis* (*Sc*) SdhA and *W. succinogenes* FrdA subunits. Only the region surrounding alanine 86 is shown.

SUPPORTING REFERENCES

Lancaster, C.R.D., Groß, R., and Simon, J. (2001) A third crystal form of *Wolinella succinogenes* quinol:fumarate reductase reveals domain closure at the site of fumarate reduction. *Eur J Biochem* **268**: 1820-1827.

Palmer, T., Sargent, F., and Berks, B.C. (2005) Export of complex cofactor-containing proteins by the bacterial Tat pathway. *Trends Microbiol* **13**: 175-180.

Simon, J., Groß, R., Ringel, M., Schmidt, E., and Kröger, A. (1998) Deletion and site-directed mutagenesis of the *Wolinella succinogenes* fumarate reductase operon. *Eur J Biochem* **251**: 418-426.

Simon, J., Groß, R., Klimmek, O., and Kröger, A., (eds) (2000) *The Genus Wolinella.* New York: Springer-Verlag.
